# Inflammation, Innate Immunity, and the Intestinal Stromal Cell Niche: Opportunities and Challenges

**DOI:** 10.3389/fimmu.2015.00319

**Published:** 2015-06-18

**Authors:** Benjamin M. J. Owens

**Affiliations:** ^1^Translational Gastroenterology Unit, Experimental Medicine Division, Nuffield Department of Clinical Medicine, John Radcliffe Hospital, University of Oxford, Oxford, UK; ^2^Somerville College, University of Oxford, Oxford, UK

**Keywords:** stromal cells, stromal immunology, intestinal inflammation, intestinal stromal cells, chronic inflammation, intestinal innate immunity

## Abstract

Stromal cells of multiple tissues contribute to immune-mediated protective responses and, conversely, the pathological tissue changes associated with chronic inflammatory disease. However, unlike hematopoietic immune cells, tissue stromal cell populations remain poorly characterized with respect to specific surface marker expression, their ontogeny, self-renewal, and proliferative capacity within tissues and the extent to which they undergo phenotypic immunological changes during the course of an infectious or inflammatory insult. Extending our knowledge of the immunological features of stromal cells provides an exciting opportunity to further dissect the underlying biology of many important immune-mediated diseases, although several challenges remain in bringing the emerging field of stromal immunology to equivalence with the study of the hematopoietic immune cell compartment. This review highlights recent studies that have begun unraveling the complexity of tissue stromal cell function in immune responses, with a focus on the intestine, and proposes strategies for the development of the field to uncover the great potential for stromal immunology to contribute to our understanding of the fundamental pathophysiology of disease, and the opening of new therapeutic avenues in multiple chronic inflammatory conditions.

## Stromal Cells and Stromal Immunology

“Stroma” has a word origin in the late Latin *strōma*, meaning mattress or covering, and is derived ultimately from the Greek word for bed. Thus, stroma is used within medicine as a term to describe the structural or connective tissue of organs, comprised of cells that act in a supportive capacity to the parenchymal cells performing specific organ function. The term “stromal cells” is often used in a confusingly broad sense in the literature, but for the purposes of this review, it refers to non-hematopoietic (i.e., CD45^−^), non-epithelial (i.e., EpCAM^−^) cells that are not of endothelial origin (i.e., CD31^−^), including all fibroblast and myofibroblast populations, as well as “immunological” stromal cell subsets analogous to those found in lymphoid tissue (Table 1).

KEY CONCEPT 1. Stromal Immunology An emerging field of immunology research that focuses on illuminating the diversity of responses mediated by non-hematopoietic, non-epithelial cells during immune responses.

**Table 1:** **Hematopoietic, epithelial, and stromal immune cells of the intestine**.

	Hematopoietic	Epithelial	Stromal
Defining markers	CD45^+^ (+ lineage specific)	CD45^−^ EpCAM^+^ Villin^+^ E-cadherin^+^	CD45^−^ EpCAM^−^ Collagens
**Subsets?**	Multiple subsets: – Dendritic cells – Macrophages – ILCs – B cells – T cells – NK cells etc.	Multiple functionally specialized subsets: – Paneth cells – Goblet cells – M cells – Enteroendocrine – LGR5^+^ stem cells	Endothelial/Lymphatic: CD31^+^ LYVE-1^+^ “Lymphoid tissue-like”: gp38^+^ CD90^+^ ICAM-1^+^ (Myo)fibroblastic: αSMA^+^ FAPα^+^ (Other stromal cell subsets remain ill-defined)

**Ontogeny**	Derive ultimately from hematopoietic stem cells (HSCs) Specific precursors for individual subsets Cells can develop outside the intestine and traffic to the tissue or develop from precursors within the gut in some contexts	Derive locally from specialized stem cell population(s) High-turnover rate requires continual replenishment from stem cell pool in base of crypts Signals from local mesenchyme direct stem cell differentiation	Derive ultimately from mesenchymal stem cells (MSCs) Precursors unclear Limited data in intestine, but stromal cells may also arise from other sources, e.g., adipocytes or monocytes (during inflammation)

**Immune functions**	Production of cytokines, chemokines and other effector molecules Induction of host-protective immunity, via cell-intrinsic and extrinsic mechanisms Immune modulation of tissue homeostasis (angiogenesis, wound healing, etc.)	Conditioning of local immune cell function Some evidence of direct innate immune function via expression of HLA-DR and NLRs/TLRs Antibacterial function Production of cytokines that regulate immune cells locally (e.g., TSLP, TNFα, GM-CSF)	Some evidence of direct innate immune function via expression of HLA-DR and NLRs/TLRs Production of cytokines that regulate immune cells locally (e.g., IL-1β, TNFα, GM-CSF) and chemokines to localize cells to and within tissue Provide a structural framework for immune cell organization

*Immune cells of several distinct lineages are present within the intestine, and contribute to immunological functions at this tissue site. Best studied are the immune functions of hematopoietic and epithelial cells, and the development and ontogeny of these cells are now well described. By contrast, the contribution of stromal cells to immunity is less known, and multiple fundamental questions regarding these cells are still under active investigation*.

Historically, stromal cells were considered as “non-immune” cells and were relegated to merely providing a structural framework upon which conventional, hematopoietic immune cells could function. However, the last decade has revealed huge complexity in the subsets of stromal cells with direct immunological properties, as well as the diversity of their contributions to the function of the immune system (1). Nevertheless, the majority of work has focused on stromal cell populations of lymphoid tissues (e.g., Lymph Nodes and Spleen) and has mainly been restricted to studies in murine model systems. Only more recently have the immunological roles of stromal cells in other tissues begun to be investigated in detail, and a concerted effort made to translate findings from murine studies to human tissues. Despite much interest in their role within the gut, many limitations remain in our understanding of the fundamental immunobiology of stromal cells, particularly relating to their ontogeny, phenotypic characteristics, and the extent of their contribution to intestinal immune responses. It is critically important to investigate heterogeneity within the intestinal stromal cell (ISA) compartment, as there is still very little known regarding the existence of stromal cell subsets within the gut, particularly in terms of specific surface marker expression, their microanatomical localization and their individual functional properties.

## Intestinal Stromal Cells as Innate Effectors

**Figure 1:**
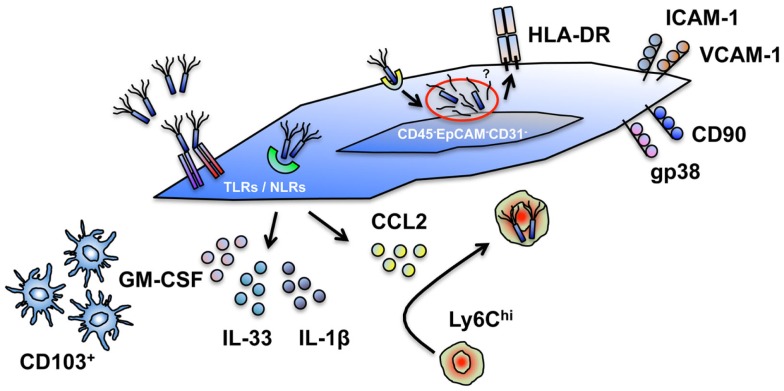
**Cell intrinsic and extrinsic innate immune functions of intestinal stromal cells**. Human and murine intestinal stromal cells (iSCs), classified as CD45^−^EpCAM^−^CD31^−^CD90^+^(*gp38*^+^*)iCAM-1^+^VCAM-1^+^ cells display a capacity for the modulation of innate immune responses via several mechanisms. (i) iSCs express a diverse repertoire of TLRs and NLRs, allowing them to directly sense intact organisms, or their products. (ii) iSCs elaborate a range of cytokines upon encounter with foreign organisms. (iii) iSC-derived cytokines (e.g., GM-CSF) can modulate the function of professional innate immune cells; iSC-derived chemokines (e.g., CCL2) can recruit professional innate immune cells. (iv) iSCs can phagocytose and internalize bacteria, and process exogenous antigen. (v) iSCs express HLA-DR and can functionally modulate effector and regulatory T cell populations. The innate functions of iSCs are – in general – less efficient than in myeloid cells, and lead to the designation of iSCs as “non-professional innate immune cells.” *Currently only determined in murine systems.

Far from being merely passive structural entities, stromal cell populations exhibit a capacity for diverse cell-intrinsic and -extrinsic immune function in many non-lymphoid tissues, including the intestine (2). These newly appreciated immune functions are reminiscent of those now well described for intestinal epithelial cells, which for a long time were also considered to be “non-immune” cells (3). Distinct from both epithelial and hematopoietic cell lineages (Table 1), stromal cells are a heterogeneous group of cells derived from mesenchymal progenitors that constitute a major component of the intestinal mucosa (4). We recently revealed a mechanism for innate sensing of pathogenic bacteria by primary human colonic CD45^−^EpCAM^−^CD31^−^CD90^+^ stromal cells (5), supporting previous data derived from human iSC lines (6) and murine infection models (7) that together suggest that tissue stromal cells can act as sentinel innate immune populations within the intestine. In line with others, we proposed that iSCs are designated as “Non-professional innate immune cells” (Figure 1) (5, 8–10). This reflects their capacity for innate sensing of bacteria (and possibly other organisms) via TLRs/NLRs, their potential to express HLA-DR/MHCII, and their ability to support/modulate the function of T cell populations, including regulatory T cells (Tregs) (11). Despite these cardinal features of innate immune cell populations, it is clear that the functional ability to take up, process and present foreign antigen is more limited for iSCs than in prototypic “professional” innate immune cells, such as macrophages, dendritic cells, and monocytes (5, 9). In addition, levels of innate immune (TLR) receptor expression in iSCs appear to be lower than in epithelial cell populations from the human intestine (12).

KEY CONCEPT 2. Non-professional innate immune cells The concept that intestinal (and other tissue) stromal cells can exhibit innate immune functions (PRR expression, pathogen-induced cytokine production, antigen processing, and antigen presentation) but in a more limited capacity than professional (myeloid) innate immune cells.

Such a designation does not, however, preclude iSCs from playing an important and active innate immunological role within the intestinal mucosa. iSCs are located at strategically important positions within the gut, immediately adjacent to the intestinal epithelium and in direct contact with the blood vasculature and lymphatic network (4). Thus, they are perfectly positioned to act as rapid “first responder” innate sentinel cells in the event of epithelial breach and bacterial translocation from the gut lumen, or after an encounter with an invasive bacterial species such as *Salmonella* (Figure 1).

KEY CONCEPT 3. iSCs as “first responders” The concept that iSCs can act as rapid-acting sentinels that sense bacterial (or other) challenge in the gut as a result of epithelial layer breach, or infection with an invasive pathogen.

As they are equipped with various mechanisms to directly sense bacterial contact (5–7), stromal cells are able to respond rapidly to local contact with a pathogen and elaborate a range of processes to further coordinate a protective immune response, as well as responding to cytokine signals from the epithelium and thus amplify both protective – and potential deleterious – immune responses. As chemokine production is a major feature of stromal cell biology in lymphoid organs (1), and iSCs are a critical source of chemokines during bacterial infection *in vivo* (7), their ability to recruit, retain, and functionally modulate professional innate immune cell populations at the site of an infection is likely to be a major component of the protective immune function of iSCs. Indeed, recent work has revealed a direct role for GM-CSF production by stromal cells of the murine small intestine in conditioning local dendritic cell function (13), supportive of our finding that *CSF2* expression is increased rapidly upon sensing of *Salmonella* by human iSCs (5). GM-CSF is also known to regulate several parameters of myeloid cell function during colitis – including the expansion of myeloid precursors within the gut (14) – highlighting that cell-extrinsic iSC function may also play a role in regulating mucosal defense via interactions with “professional” myeloid APC populations.

KEY CONCEPT 4. iSCs as amplifiers of immune responses The concept that iSCs integrate signals from other cell types (epithelial, hematopoietic, endothelial) and produce factors that amplify immune responses during intestinal infection or inflammation.

Furthermore, as iSCs are known to have some phagocytic capacity (5) and stromal cells of other organs are able to induce pathogen eradication pathways such as the production of nitric oxide (15, 16), it remains possible that iSCs also play a role in limiting infections of the intestine via cell-intrinsic antimicrobial effector mechanisms.

Taken together, these emerging data suggest that iSCs are likely to play an important adjunct role in the defense of the intestine from mucosal pathogens. However, as these observations were mostly made using *in vitro* experimental approaches with *ex vivo* cultured cells; further work is required in order to fully validate their veracity.

## Dissecting Stromal Innate Immune Response Relevance *In vivo*

Definitively proving a role for stromal innate sensing within the intestine *in vivo* remains challenging. Recent work utilizing irradiation bone marrow chimeric approaches defined a major role for the expression of NLR family members – and concomitant inflammasome activation – in non-hematopoietic cells of the murine intestine (17, 18). Despite the authors’ conclusion that these cells were epithelial, there remains a possibility that iSCs – also a radioresistant population – may play a role. This is supported by observations that murine (19) and human (5) colonic stromal cells express NLR family members such as NLRP3 and NLRP6, thus making it difficult to exclude a role of stromal cells in the innate sensing and cytokine production process *in vivo* solely using such chimeric approaches.

The current “gold standard” approach to elucidating the role of specific protein expression by individual cell types during immune responses *in vivo* is to use c*re-Lox* technology that allows for ablation of target protein mRNA expression under the control of a cell-specific promoter. This is currently feasible for intestinal epithelial cells using the Villin-*cre* system (20), but strategies for the ablation of target protein expression specifically in iSCs remain elusive. Nevertheless, attempts to modulate iSC function have been made using this technology. Ablating TNFα receptor expression by intestinal mesenchymal cells using Collagen 6 (*ColVI)-cre TnfRI^fl/fl^* mice revealed an essential role for stromal cells in the development of the Crohn’s-like TNFΔARE model of ileal inflammation (21), and similar studies using *ColVI-cre* mice have elucidated roles for stromal cells in other inflammatory intestinal contexts (22). In addition, a recent study using the *ColVI-cre* system ablated MyD88 expression in gut stromal cell populations, reporting a defect in PD-L1 expression by these cells during DSS colitis *in vivo* that was proposed to regulate mucosal IFNγ expression and thus contribute to disease (10). Although complicated by the shared role of MyD88 in both IL-1 and TLR signaling pathways, this is the first formal suggestion of a potential role for TLR sensing mechanisms within the intestinal stromal compartment regulating mucosal inflammatory responses *in vivo*.

However, as *ColVI* expression (and thus *cre-*recombination) is detected in stromal cells of the joint, skeletal muscle, skin, intestine, and heart in these mice (21), the conclusion that such a genetic strategy reflects a phenotype as a result of specific gene deletion solely within the intestinal stromal compartment is most likely an oversimplification. In order to reach a more refined answer on the role of specific innate sensing pathways, strategies must be developed that allow for the ablation of gene expression only within iSCs. However, limitations in our understanding of the fundamental biology of these cells – particularly a lack of cell-specific markers – remain a major hurdle to overcome in achieving this goal.

KEY CONCEPT 5. iSCs lack specific surface marker expression A major avenue for future research relating to the paucity of specific (surface) marker expression by iSCs that limits our ability to identify iSCs in tissue, and to use sophisticated genetic strategies to modulate their function *in vivo*.

Podoplanin (*Pdpn;* gp38), already known as a marker of several stromal cell subsets of the lymphoid organs, is emerging as a promising marker of iSCs, at least in murine systems (13, 23, 24), and a *Pdpn-cre* murine strain has been developed (25). Thus, it is at least conceptually feasible to reach a position whereby innate immune receptors/key innate signaling modules are ablated specifically in gp38^+^ stromal cells and the effects on an intestinal infectious or inflammatory insult determined. However, as gp38 is expressed widely by stromal cell populations, particularly in lymphoid tissues (1, 26), the problem remains that any observed effects on intestinal pathology may be due to the secondary effects of ablating innate immune functionality in gp38^+^ stromal cells outside the gut. Although studying stromal cell ontogeny *in vivo* remains challenging, a murine tool based on expression of Platelet-derived growth factor-α (*Pdgfra*) has now been developed to allow for lineage tracing of stromal cell populations within the skin (27). This approach revealed significant heterogeneity within stromal populations at this barrier surface, and represents a powerful tool for studying the ontogeny and function of stromal cell populations *in vivo*. As yet, no similar tool has been validated for use in the intestine, and thus extensive further work – including the single-cell transcriptional profiling of stromal cell subsets from the intestine (and other organs) – is essential in order to identify novel, lineage-specific markers that will allow for the specific function of these cells to be interrogated *in vivo*.

## Imprinted Responses and Trained Innate Immune Memory

A fascinating aspect of stromal cell biology is the observation that stromal cells of multiple tissues exhibit stable, imprinted phenotypic changes as a result of chronic inflammation. This has been extensively studied in synovial stromal cells from the joints of rheumatoid arthritis (RA) patients (28), which may reflect specific effects of inflammation in this highly specialized tissue microenvironment that – unlike the intestine – is not routinely exposed to bacterial products. However, similar stable alterations in tissue stromal cell phenotype and function have been reported in several organ systems (29, 30), including the intestine (31, 32). The mechanisms by which stromal cells contribute to this chronic inflammatory microenvironment are likely to be diverse, and include the provision of inflammatory cytokines, the recruitment, retention, and activation of hematopoietic immune cells, and pro-inflammatory crosstalk with other tissue cells, such as the vascular endothelium (28, 33).

KEY CONCEPT 6. Stromal cells exhibit imprinted functional changes in inflammation The concept that stromal cells of many tissues are activated in inflammation and retain this activated phenotype upon *ex vivo* isolation from tissues and through multiple passages in *in vitro* culture. Epigenetic modifications around key gene loci likely mediate these stable changes.

Although less well characterized than their synovial counterparts, stromal cells of the intestine appear to undergo persistent functional changes in chronic disease. A recent murine study revealed that cultured colonic iSCs exhibit enhanced and sustained expression of the decoy cytokine receptor soluble ST2 (sST2) during active bacterially driven colitis; a phenomenon proposed to contribute to disease by limiting the bioavailability of regulatory T cell-inducing IL-33 (34). Although it is not known whether these functional alterations are a direct result of bacterial sensing by iSCs, increased levels of sST2 are found in the sera of patients with active ulcerative colitis (35) – a condition associated with significant bacterial translocation and, most likely, stromal cell activation.

Evidence is now building that suggests epigenetic modifications within synovial and other tissue stromal cells due to chronic inflammation underlie their persistently activated phenotype (30, 36–39). Epigenetic changes in synovial stromal cells vary depending upon the stage of disease (early vs. late RA), and the location of altered DNA methylation sites within the genome are also distinct between disease stage (40). Inflammatory cytokines can induce epigenetic changes in synovial stromal cells (41), and pharmacological epigenetic modulation can alter the production of inflammatory cytokines and chemokines by stromal cells from RA patients (42). These so-called “inflammatory memories” at the level of the tissue stroma are proposed to be a major determinant in the chronicity of inflammation within the joint (43), and contribute to the conceptual framework that persistently activated stromal cells provide a “fertile soil” for the propagation of chronic inflammatory disease (44).

**Figure 2:**
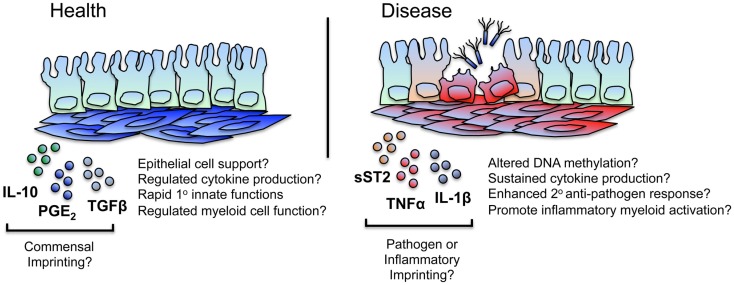
**iSCs may exhibit imprinted regulatory and inflammatory immune responses**. iSCs can produce both pro- and anti-inflammatory cytokines and elicit both activatory (innate host-protective) and regulatory immune responses. Based on stromal cells of other tissues, this may include the “tolerogenic” regulation of myeloid cell function in the healthy gut, mediated via stromal cell products such as IL-10, PGE_2_, and TGFβ. Such tonic signaling responses may be as a result of conditioning by normal commensal flora – either directly or via the epithelium – with iSCs retaining a “memory” of these regulatory signals that may break down or be superseded during chronic inflammation or after an encounter with a pathogen. During chronic inflammation in mice, iSCs produce enhanced levels of mediators such as sST2, regulating the bioavailability of regulatory T cell supporting cytokines and likely contributing to disease. Based on observations in other chronic inflammatory conditions (such as RA), iSCs are a likely source of many pro-inflammatory mediators, and may exhibit alterations in their epigenetic status. This may result in sustained cytokine and chemokine production, thus amplifying inflammation (e.g., via myeloid cells) at the site of local tissue inflammation. iSCs may also retain a “trained memory” of a pathogen encounter, and as such display enhanced protective responses to a secondary encounter with the same, or a different, pathogen. Such responses may be aberrantly induced during chronic inflammation.

Further work is required to unravel the extent to which persistent inflammatory changes occur in iSCs during intestinal inflammation – as well as the mechanisms underlying these changes – but given similarities in the pathophysiology of RA and inflammatory bowel disease (IBD), it is highly likely that epigenetic imprinting of pro-inflammatory functions also occurs within intestinal tissue stromal cell populations during IBD (Figure 2).

How could these observations of persistent and stable tissue stromal cell activation during inflammation relate to innate immune function? A potential answer comes from a recently proposed phenomenon: “*trained immunity*” (45, 46). This new conceptual framework has many similarities to the hypothesis that links persistent inflammation with stromal epigenetic changes as discussed previously. Breaking the paradigm that restricted immunological memory to adaptive immune populations such as T and B cells, pioneering recent work has uncovered the molecular basis of a mechanism by which innate immune cell populations retain a memory of previously encountered pathogenic organisms, leading to enhanced host defense upon secondary pathogen encounter. With data currently restricted to observations in natural killer (NK) cells and macrophage subsets, this process is mediated by altered methylation and acetylation patterns around critical functional gene promoters (47) and is associated with fundamental changes in innate immune cell metabolism (48).

Such a framework is highly appealing when considering the potential effects of persistent stromal cell activation on protective innate immune responses. With a far slower turnover rate than intestinal epithelial and hematopoietic immune cells [100–130 days in mice (49)], gut tissue stromal cells are primary candidates for a cell type capable of integrating – and retaining memory of – signals that allow for a modulated secondary functional response. Thus, in addition to stable changes imprinted in iSCs as a result of non-specific inflammatory processes, it is reasonable to hypothesize that stable changes in stromal cell function may occur after iSC encounter with a (non-cytolytic) pathogen. The types of functional responses may be the same as or differ from those induced solely by inflammatory stimuli, vary between the type of pathogen encountered, and lead to enhanced cell-intrinsic or -extrinsic innate immune functions. Interestingly, it would appear that trained immunity in macrophages can induce both activated and suppressive immune responses (50). Similarly, stromal cells of many tissues can induce immunoregulatory responses alongside inflammatory processes (10, 51), and so it is feasible that inherently tolerogenic mechanisms of stromal cells within the healthy intestine may be linked to a “memory” of contact with quiescent bacterial species (or their products) from the normal intestinal flora (Figure 2).

While the cellular longevity of tissue stromal cells is also pertinent to the inflammatory contexts discussed previously, it adds significant weight to an argument that proposes iSCs as the cell type responsible for trained immunity within the intestine, placing them as tissue resident, long-lived, multifunctional innate immune cells capable of retaining functional memories of an encounter with pathogenic organisms – responses that may be dysregulated and subsequently contribute to chronic inflammation. However, this hypothesis is currently highly speculative, and whether such processes do indeed occur in iSCs, the functional diversity of any responses elicited and the mechanisms underlying them remain to be determined.

KEY CONCEPT 7. iSCs as mediators of “trained immunity” in the intestine? A hypothetical framework positing that iSCs are a cell type responsible for retaining a “memory” of encounters with pathogenic organisms in the gut, allowing them to mediate protective responses to secondary encounter with the same or a different pathogen. Potentially mediated via epigenetic and/or metabolic reprograming.

## Stromal Immunology: A Call to Action

Although hampered by a lack of tools to assess stromal function, particularly *in vivo*, the wealth of data rapidly emerging in the stromal immunology field strongly argues for a concerted effort in bringing the study of these cells to equivalence with their hematopoietic immune cell cousins. Large studies have recently identified that the stromal cell compartment of tumors drives progression and poor clinical outcome in colorectal cancer patients with aggressive disease (52, 53), indicating that a deeper understanding of the biological functions of the iSC niche may also yield exciting potential therapeutic strategies in other disease indications. The diversity of potential stromal cell functions in both innate immune defense and inflammatory disease highlights the need to dissect in more detail their functionality during immunological processes, and supports their designation as “non-hematopoietic immune cells.” Rapid progress has been made in the acceptance of stromal cells as a key component of immune responses, but in order to fully validate them as direct contributors to these processes, advanced tools must be developed to interrogate their function in a sophisticated manner. Only by doing this will the full contribution of these fascinating cells to immunology be revealed.

## Conflict of Interest Statement

Benjamin M. J. Owens is supported by an Oxford – UCB Pharma Postdoctoral Fellowship from The University of Oxford and UCB Pharma Ltd. UCB Pharma Ltd. had no role in the conception, writing, or editing of this manuscript.
